# The effect of a Computerised Decision Support System (CDSS) on compliance with the prehospital assessment process: results of an interrupted time-series study

**DOI:** 10.1186/1472-6947-14-70

**Published:** 2014-08-09

**Authors:** Magnus Andersson Hagiwara, Björn-Ove Suserud, Boel Andersson-Gäre, Bengt- Arne Sjöqvist, Maria Henricson, Anders Jonsson

**Affiliations:** 1 University of Borås, School of Health Sciences, 501 90 Borås, Sweden; 2 School of Health Sciences, Jönköping University, Jönköping 551 11, Sweden; 3 Department of Signals and Systems, Chalmers University of Technology, Gothenburg, Sweden

## Abstract

**Background:**

Errors in the decision-making process are probably the main threat to patient safety in the prehospital setting. The reason can be the change of focus in prehospital care from the traditional “scoop and run” practice to a more complex assessment and this new focus imposes real demands on clinical judgment. The use of Clinical Guidelines (CG) is a common strategy for cognitively supporting the prehospital providers. However, there are studies that suggest that the compliance with CG in some cases is low in the prehospital setting. One possible way to increase compliance with guidelines could be to introduce guidelines in a Computerized Decision Support System (CDSS). There is limited evidence relating to the effect of CDSS in a prehospital setting. The present study aimed to evaluate the effect of CDSS on compliance with the basic assessment process described in the prehospital CG and the effect of On Scene Time (OST).

**Methods:**

In this time-series study, data from prehospital medical records were collected on a weekly basis during the study period. Medical records were rated with the guidance of a rating protocol and data on OST were collected. The difference between baseline and the intervention period was assessed by a segmented regression.

**Results:**

In this study, 371 patients were included. Compliance with the assessment process described in the prehospital CG was stable during the baseline period. Following the introduction of the CDSS, compliance rose significantly. The post-intervention slope was stable. The CDSS had no significant effect on OST.

**Conclusions:**

The use of CDSS in prehospital care has the ability to increase compliance with the assessment process of patients with a medical emergency. This study was unable to demonstrate any effects of OST.

## Background

Prehospital emergency care is characterized by judgement and decision making (JDM) in an unstable setting [1]. Prehospital clinicians have had to make advanced medical decisions a long distance from medical support, they have to assess and treat patients with many different symptoms and conditions in altered settings. The typical ambulance mission can be divided in 7 phases; receiving the call, arriving at the address, performing an on-scene assessment, performing an initial patient assessment, transport the patient to the ambulance, performing additional assessment and treatment on route, arriving to the hospital and handing over the patient [1]. Research has suggested that it is in the on-scene phase of an ambulance mission that imposes the greatest demands on the prehospital clinicians JDM process. It is outside the ambulance the clinicians have access to limited cognitive support and have to deal with varying settings [2]. In a case study with the aim to investigate the use of guidelines and protocols among prehospital clinicians in Sweden [3], the greatest obstacle to the use of guidelines was their physical format, which was due to the development process, which produces guidelines that are not suited to use in the prehospital setting. The consequence of the poor format were that the guidelines were seldom used explicit in the on scene patient assessment [3].

The limited research in prehospital patient safety suggests that most important issues related to patient safety is errors in the JDM process among prehospital clinicians [4]. Except the unstable settings were the prehospital care is execute, there can be several reasons to poor JDM processes. One suggestion is that prehospital clinicians not always are supported with right tools or that the education is not adequate for the complex work [5]. Other reasons could be lack of feedback [6], fatigue, stress [7], motivation and morale [8]. The use of cognitive aids such as guidelines, protocols, checklists and algorithms is considered as important in the execution of safe prehospital care [9]. However, there are studies that suggest that compliance with guidelines in some cases is low in the prehospital setting [10-14]. There can be several reasons for poor compliance with guidelines. The implementation strategies are one possible cause [15], while factors such as guideline visibility, content, design and the level of evidence have been shown in other health-care settings to have an impact on compliance with guidelines [16].

In in-hospital emergency settings, computerised decision support have been proved to have the ability to increase compliance to guidelines and processes of care [17]. Four features have been identified as important factors for the ability of decision support systems to improve clinical practice; the decision support is computer based, gives support as part of natural workflow, the decision support is delivered at the time and location of the decision making and actionable recommendations are provided [18]. There is limited evidence relating to the effect of CDSS in a prehospital setting [19]. In a recent simulation study of a CDSS effect of compliance to guidelines and CDSS effect of on-scene time (OST), improved guideline compliance was found among the ambulance clinicians who used a CDSS in two simulated patient cases compared with those that used guidelines in the usual paper format, but the group using the CDSS also spend more time on-scene [19].

Most studies of guideline adherence in prehospital care have studied adherence to single diagnosis and treatment plans. Then bias in the JDM process seems to be the major threat against patient safety in prehospital care; we aimed to evaluate the CDSS effect on the basic assessment process of the patient with medical emergencies.

A poorly designed CDSS can potentially produce harm. For examples a poorly designed interface that are unclear or irrational can results in error even among computer experience users [20]. A poorly design CDSS in prehospital care could results in an increased on-scene time (OST). In some emergencies, the time spent on-scene can negatively affect patient outcome [21]. The second aim was therefore to measure the OST time, defined as the time when the ambulance arrives at the address to the time when transportation is initiated.

## Methods

### Setting and participants

The study was conducted at a single ambulance station in south-west Sweden. The station’s catchment area covers both rural and urban areas. Most of the patients are transported to the nearest hospital, which is located 37 kilometers from the ambulance station. The ambulance undertook approximately 1900 missions in 2012. The entire regular employed at the station participated in the study. The regular employed comprises nine ambulance nurses (AN) and one emergency technician (EMT). Of them six person are male and four female. The average age is 45.9 years (range 34-60) and the average experience from prehospital care is 16.9 years (range 2-35). Patients with symptoms of the most common medical emergencies described in the local prehospital guidelines were eligible for the study. This included patients with symptoms of chest discomfort, breathing problems, neurological symptoms, allergic symptoms, abdominal pain, affected circulation, including failing heart-conducting system, affected general condition, infections and endocrine system symptoms.

The exclusion criteria were:

Patients < 18 years.

Patients suffering from trauma or poison.

Patients transported between hospitals.

Patients who were pregnant.

Patients with no circulation and breathing.

The exclusion of patients who were pregnant or had some kind of trauma was due to the fact that, at this stage, the CDSS did not deal with these problems. A demographic description of the patients who were included can be found in Table 1.

**Table 1:** Study objects characteristics and disposition

**Variable**	**Pre-Intervention (n =175 )**	**Post-Intervention (n =196 )**	**p-value**
Age, years, mean (SD)	66.7 (19.9)	69.7 (20.5)	0.04
Sex, male (%)	43.4	54.1	0.04
Sex, female (%)	56.6	45.9	
Diagnostic category			
Cardiovascular symptoms (%)	19.4	21.9	0.86
Respiratory symptoms (%)	17.7	14.3	
Neurological symptoms (%)	28.0	29.6	
Gastrointestinal symptoms (%)	15.4	15.8	
Effected general conditions (%)	2.3	1.5	
Effected circulation including failing heart conducting system (%)	9.1	7.1	
Infections (%)	4.6	6.6	
Endocrine system symptoms (%)	1.7	2.0	
Allergic symptoms (%)	1.7	0.5	
Number of patient assessed per ambulance personnel			
Ambulance personnel 1	17	24	0.29
Ambulance personnel 2	20	14	
Ambulance personnel 3	15	16	
Ambulance personnel 4	16	27	
Ambulance personnel 5	26	23	
Ambulance personnel 6	17	23	
Ambulance personnel 7	16	19	
Ambulance personnel 8	18	19	
Ambulance personnel 9	13	11	
Ambulance personnel 10	17	10	

The significance of continues variables was based on independent T-test and categorical variables Chi-square tests.

A more detailed context description can be found in a previous study [3].

### Intervention

The evaluated intervention was a Computerized Decision Support System (CDSS) designed for prehospital care. The CDSS can be defined as an expert system [22] and guides the prehospital clinicians through a systematic assessment process based on the content of the Advanced Medical Life Support (AMLS) system [23]. The CDSS was handheld (Panasonic Toughbook) and used the MobiMed 4.0 pre-hospital eHealth platform from Ortivus AB. The prehospital clinicians were trained to use the CDSS when they performed the patient assessment. The CDSS consists of four main areas.

#### First survey

The goal of the first survey is to assess and treat life-threatening conditions. It starts with an assessment of the airways and continues with an assessment of breathing, circulation and disability. During the assessment, the ambulance clinicians can also choose to receive additional support when it comes to assessing and treating problems in the different areas by using optional algorithms. For example, when treating a difficult airway, the CDSS provides an optional algorithm over airway management in 5 steps.

#### History

When the first survey is finished, the CDSS continues with focused history collection.

The CDSS guides the prehospital clinicians through a battery of questions. The questions start with signs and symptoms and continue with questions relating to onset, palliation, quality, radiation, severity, time, allergies, medication, past medical history, last oral intake and events prior to illness.

#### Chief symptoms

After finishing the history collection, the prehospital clinicians have to choose the patient’s chief symptom. Examples of symptoms are chest discomfort, breathing problems, abdominal pain and altered mental status. When a chief symptom has been chosen, the CDSS provides a list of further focused medical assessments based on the symptom.

#### Field diagnosis

The last page in the CDSS is field diagnosis. Here, the CDSS provides a list of field diagnoses based on the chief symptom. Every field diagnosis is linked to the local prehospital guidelines where the ambulance staff can obtain information about diagnoses and also obtain access to treatment plans. The CDSS is also linked to a medical record system. All the actions performed in the CDSS are documented in the system. The documentation comprises ticking boxes and free text fields. When the patient assessment is complete, a medical record can be printed out for use in the hand-over phase.

Unfortunately, we were not able to configure the CDSS with the ordinary prehospital records system. The study participants had to make the first documentation in the CDSS and, later at the hospital, new documentation was created on a stationary computer in the Emergency Room (ER).

Apart from using the CDSS in the assessment of the patient, the participants were instructed to work as usual.

### Study design and outcome measures

The study had a two-phase time-series intervention design [24], where study data were collected on a weekly basis during the study period. The Quality Criteria for Interrupted Time Series (ITS) Designs [25] checklist was used as a guide.

The study was conducted from November 2012 to May 2013. November, December, January made up the baseline period, when the clinicians worked as usual, supported by paper-based guidelines. The usual guidelines are conducted of three different systems; 1) the main guideline which contains description of assessment of the medical and trauma patient. It also contains description of different symptoms and conditions, directions for treatments and pathway protocols. The main guideline is a file with 194 A4 pages and is located between the seats in the front of the ambulance and one copy in the back of the ambulance. 2) A pocket guideline containing tables of drug doses, normal values and a few algorithms. The ambulance clinicians usually have the pocket guideline in a leg pocket. 3) The triage protocol which is a triage guide in a text file located in the back of the ambulance. A more detailed description of the use of guidelines in the organization can be found in a recent study [3].

The intervention period was March, April and May 2013. February was not included, since training on the CDSS was conducted on three occasions that month. The training consisted of a lecture in which the CDSS functions were presented, followed by manikin training with the CDSS, for a total of four hours. Apart from the training on the CDSS, no other events which could have influenced the outcomes were identified.

The main outcome was compliance with the basic assessment of the medical patients described in the local and national prehospital guidelines [26,27], which are in turn guided by the content of the Advanced Medical Life Support (AMLS) system [23]. The second outcome was OST, defined as the time when the ambulance arrives at the address to the time when transportation is initiated. The two outcomes were compiled weekly during the study period. The objective of assessing the outcome weekly is dependent on the fact that, during the course of a week, six to eight of the 10 participants were on duty and expected individual differences should even themselves out during a week. As the study was ongoing for six months and there was one data point every week, the study had 24 data points.

Since power calculation is difficult in time series, no power was calculated. Instead a power calculation from a simulation study [28] was used. The simulation-based power calculation displayed that with 12 data points in the pre-intervention period and 12 data points in the post-intervention period, there is more than 80% chance to obtain an effect size of 1.0 or more. The power is increased when the effect size is increased. The power are also dependent of the degree of auto correlation there data with a low degree of auto correlation have a higher power [28].

A rating protocol was developed for the study (see Additional file 1). The protocol was based on the description of how to assess patients with medical emergencies in the local and national guidelines [26,27]. The protocol consists of 33 assessment interventions and a protocol in which all 33 interventions were completed was defined as 100% compliance. Two of the authors, (MAH, BOS) was pilot-tested the rating protocol. They separately rated the same 10 records and their interrater reliability (IRR) was calculated using Cohen’s k. The IRR was found to be = kappa 0.75 (p = 0.001) which is considered as a substantial agreement [29].

Eligible patients for the study were searched for in the ambulance organization’s patient record database, according to directions for inclusion and exclusion criteria. Patient records from included patients were extracted by one of the authors (MAH). We estimate that the extraction process identified 90 to 100% of the eligible patients.

The final rating was performed by one of the authors (MAH) who was reading the eligible ambulance patient records and identified information which describes assessment interventions included in the rating protocol. Interventions not described in the patients’ records were regarded as not having been performed. Since the primary variables were not entirely objective, the rater was blinded to the period to which the record belonged. Two co-authors (AJ, BOS) removed the date of the included records and, using a coding process, the rated records could be put in the right time period prior to analysis. The records were also randomized in time periods, as we expected the rating to change with time.

The information in the CDSS was not collected, since we wanted the same data sources in the baseline period and the intervention period. The participants in the study were aware of data collection in both the pre- and post-intervention phases.

### Analysis

For demographic data, descriptive statistics were used. To determine the distribution of eligible patients between the pre- and post-intervention phase, an independent T-test was used for continuous data and chi-square tests for categorical data.

To analyze the difference between the pre- and post-intervention phase, the regression was tested for autocorrelation in the residuals using the Durbin-Watson test.

A segmented regression analysis was performed to determine the level and slope in the pre-intervention phase and the change in level and slope in the post-intervention phase [24] on the mean percentage compliance and mean OST. The full regression model included changes in slope in the pre- and post-intervention phase and changes in level after the introduction of the CDSS. For the most parsimonious model, non-significant variables were removed stepwise for entry in the model with P-in 0.05 and P-out 0.10.

A p-value of < 0.05 was considered significant in all statistical tests.

All analyses were performed using SPSS 21.0 (SPSS, Inc, Chicago, IL).

### Ethical issues

One ethical issue when it comes to the collected design is that a limited number of individuals are closely evaluated. There is a risk that the participants may feel criticized in their professional role. The participants were guaranteed anonymity and were told that all study data would be treated confidentially. Informed written consent was obtained from all the participants in the study and they were informed that they were free to withdraw from the study at any time and that the study data relating to that participant would be erased.

The study was approved by the Regional Ethics Committee, Gothenburg, Sweden (Dnr: 1133-11).

## Result

A total of 371 patients were included in the study. There was a difference in the distribution between the pre- and post- intervention phases according to gender and age. The patients in the post-intervention phase were two years older on average compared with those in the pre-intervention phase (*p =* 0.04) and there were significantly more male patients in the post-intervention phase (*p =* 0.04). There was no significant difference in the distribution of the diagnostic categories between the phases (*p =* 0.86). There was also no significant difference in the distribution of eligible patients per participating member of prehospital clinicians in the pre- and post-intervention period (*p =* 0.29) (Table 1).

The single data points (week) comprised the ratings of 15.52 (mean) medical records, with a range of 9 to 29 records. The test for autocorrelation revealed that no autocorrelation was present. The Durbin-Watson was found to be 2.118 when the dependent variable is “mean percentage of compliance” and 2.074 with “OST” as the dependent variable. The level for no autocorrelation is 1.55-2.45.

**Figure 1:**
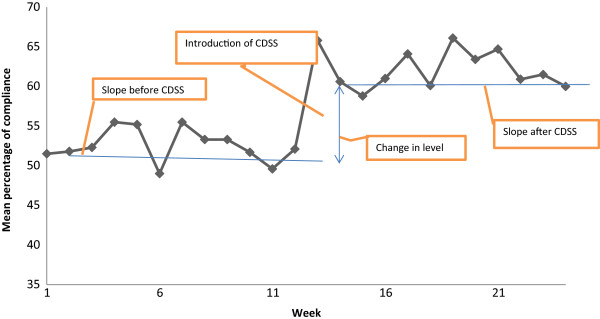
Mean percentage compliance of prehospital guidelines of the assessment process.

Just before the baseline period, the prehospital clinicians compliance with the prehospital guidelines for assessment was an average of 53% (*p =* <0.001). There was no significant change in slope in the pre-intervention phase (*p =* 0.639). After the introduction of the CDSS, there was a significant jump in the level of compliance with the assessment process. The compliance rose by 10% (*p =* <0.001). The post-intervention slope was also stable, with a non-significant change (*p =* 0.912). The most parsimonious model contained only the intercept and the change in level after non-significant parameters was removed stepwise (Table 2, Figure 1).

**Table 2:** Results of segmented regression analysis of the impact of CDSS of compliance to assessment process described in prehospital guidelines

	**Coefficient**	**Standard error**	***t*-statistic**	***P*-value**
**Full regression model**				
Intercept	53.194	1.469	36.201	< 0.001
Slope before CDSS	– 0.097	0.200	– 0.483	0.634
Change in level after CDSS	10.637	1.960	5.428	< 0.001
Change in slope after CDSS	0.031	0.282	0.111	0.912
**The most parsimonious model**				
Intercept	52.567	0.663	79.324	< 0.001
Change in level after CDSS	9.683	0.937	10.332	< 0.001

**Figure 2:**
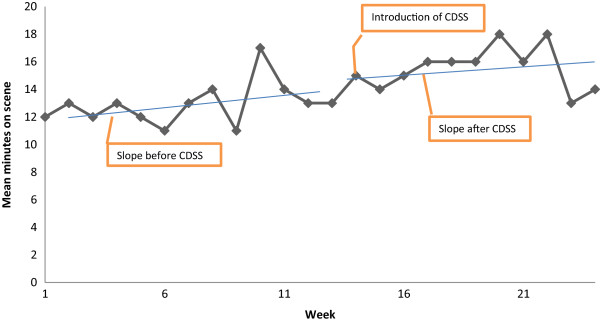
Mean On Scene Time (OST).

The introduction of the CDSS had no significant effect on the time spent on scene. The most parsimonious model contained the intercept (*p =* <0.001) and change in pre-intervention slope (*p =* <0.001). The result indicates that the OST had already increased before the introduction of the CDSS (Table 3, Figure 2). The reason for the positive pre-intervention slope is unknown.

**Table 3:** Results of segmented regression analysis of the CDSS impact of On Scene Time (OST)

	**Coefficient**	**Standard error**	***t*-statistic**	***P*-value**
**Full regression model**				
Intercept	11.712	1.001	11.703	< 0.001
Slope before CDSS	0.185	0.136	1.363	0.188
Change in level after CDSS	0.670	1.335	0.502	0.621
Change in slope after CDSS	- 0.073	0.192	- 0.382	0.702
**The most parsimonious model**				
Intercept	11.772	0.659	17.855	< 0.001
Slope before CDSS	0.188	0.046	4.080	< 0.001

## Discussion

The result of the present study indicates that the use of the CDSS can increase compliance with the basic assessment process described in the prehospital guidelines. The clinical study confirmed the result of a previous simulation study of the same CDSS. In the earlier simulation study the use of the CDSS increased the compliance to guidelines in two simulated patient cases from 60% compliance in the control group to 80% in the CDSS group. The greatest difference in compliance was in the first assessment (ABCD) and in the anamnesis of the patient [30]. The greatest advantage of the CDSS in the prehospital setting is probably the format. In a case study of the use of guidelines and protocols in a prehospital organization, the format of the guidelines was the main obstacle to using the guidelines. In general, the prehospital clinicians were in favor of using the guidelines and they regarded the guidelines as an absolute necessity, but the paper format makes the guidelines difficult to use in patient assessments [3]. The visibility and usability of the guidelines have also been identified as important features for guideline use in other settings [31].

As an effect of improved compliance with the guidelines, the prehospital clinicians performed more assessments and interventions when using the CDSS compared with the pre-intervention phase. How can these results have an effect on patient safety issues in the prehospital setting? The single most common type of human error is leaving out necessary task steps [32]. There are several reasons for the omissions; informational overload, the procedural steps are not entirely logical, premature exits in which procedures at the end of a task are left out and unexpected interruptions in the task process [32]. In a previous study, the ability to collect and use information was one of the greatest differences between expert paramedics and novices. The expert paramedics also performed more interventions and assessments compared with the less experienced paramedics [33]. An accurate prehospital assessments have been found to benefit patients with stroke [34] and ST-elevation myocardial infarctions [35]. The early prehospital identification of the symptoms shortens the time to definite care and consequently reduces mortality and complications. The ability to collect useful information and organize the information in order to sort useful information from irrelevant information is also a characteristic of the expert nurse [36]. Experienced clinicians often use schedules based on previous experience to organize information collection [36]. The CDSS in the present study can be defined as a schedule of this kind and could have the potential to reduce cognitive overload and avoid “short cuts” in the decision making process. Good clinical information systems can have a positive effect on clinical reasoning. Physicians who start using an Electronic Health Record (EHR) change the way they make decisions compared with using paper-based health records. After using the system, the physicians were more focused on problem solving with simple propositions. The cognitive changes were sustained even after the study [37]. The reason for the cognitive change is probably the way the information is processed, stored and presented [36].

However, information systems can also have negative consequences. Patel et al. [38] argue that CDSS mediate human performance. There is a risk, for example that the CDSS will slow down the development of changes in knowledge and skills [38]. There is differences in how expert and novice clinicians use CDSS. Studies have shown [37] that the experienced decision maker (expert) uses the CDSS structure and is able to complete the task using the structure, while a novice uses the CDSS to prompt and gather too much irrelevant information, which results in incorrect decisions. The expert appears to use guidelines and algorithms as a problem-solving process, whereas the novice uses the same system as an educational device [37,38].

The CDSS in the present study had no significant effect on OST, in spite of the fact that more assessments and interventions were performed. This result is in line with a previous study which reveals no relationship between the number of performed interventions and OST [39]. It is possible that, by organizing the assessment process in a CDSS, the ambulance team can work in a more organized way on the scene and as a result produce more in a shorter time.

The CDSS level of usability is also an important feature, especially in the prehospital setting, which can be described as unstable. Since we were not able to configure the CDSS with the ordinary prehospital records system, the prehospital clinicians had to document patient information twice, first in the CDSS and later in the ordinary ambulance patient record system in a computer at the ED. This double documentation probably has limited effect of the OST, but potentially increase the total prehospital time, which was not measured in this study.

The present study has several limitations. Firstly, it is important to remember that the present results relate to this CDSS in this particular context. A CDSS is a complex intervention [40]. The complexity of an intervention is determined by two variables, the number of components and the level of interrelatedness [41]. In these terms, a CDSS used in a prehospital setting can be defined as having a medium level of complexity. There are relatively few components (the ambulance team and the patient), but the components have a high degree of interrelatedness (e.g. computer interface, usability, structure, user compliance, acceptance, work culture and so on.) The results of a complex intervention study can be difficult to transfer to other settings. To increase the transferability, a deep context description is important [42]. Secondly, the study was not randomized. The ITS design is a way of strengthening the “before and after study” by controlling for secular trends, cyclical effects and random fluctuations [25]. The use of the “Quality Criteria for Interrupted Time Series Designs” [25] reveal the importance of blinding the rater when it is possible. In the present study it was handle by a process were co-authors were removed the date on the included records so the rater did not know if they belong to the baseline or intervention period. Another important issue is the quality of the protocols used in the measurements. One way to strengthen the rating protocol reliability is to determine the IRR score. In the present study the IRR was calculated to kappa 0.75 (*p =* 0.001), which is considered as a substantial agreement [29]. Thirdly, the primary variable is based on data extracted from Electronic Health Records (EHR). There is a substantial risk of data loss and the information in the EHR may not entirely reflect reality [43]. Fourthly, the study did not investigate the relationship between the increase in guideline compliance and patient outcomes. Future studies should concentrate on the way increased guideline compliance in the prehospital setting can affect outcomes such as mortality, morbidity, time in hospital, complications and time to definite care.

## Conclusions

In this interrupted time-series study, the use of the CDSS increased compliance with the basic assessment process of a patient with a medical emergency described in the local and national prehospital guidelines. One effect of the increased compliance was that the ambulance staff performed more prehospital assessments and interventions, which should help to increase patient safety. There was no significant change in the time spent on scene when the CDSS was used.

## Competing interests

The authors declare that they have no competing interests.

## Authors’ contributions

MAH was designing the study, participated in the data collection, participated in the analyses of data and drafted the manuscript. BOS and AJ were participated in the design process and helped to draft the manuscript. BAS made substantial contribution to the development of the CDSS and helped to draft the manuscript. BAG and MH were participated in the drafting of the manuscript. All authors read and approved the final manuscript.

## Pre-publication history

The pre-publication history for this paper can be accessed here:

http://www.biomedcentral.com/1472-6947/14/70/prepub

## Supplementary Material

Additional file 1 Assessment elements of the medical patient (n = 33). Click here for file
