# Supplementary material for: The effect of a Computerised Decision Support System (CDSS) on compliance with the prehospital assessment process: results of an interrupted time-series study
Source: BMC Med Inform Decis Mak. 2014 Aug 9;14:70. doi: 10.1186/1472-6947-14-70 (PMC4136405; doi:10.1186/1472-6947-14-70)
Supplement: Additional file 1 — Assessment elements of the medical patient (n = 33). [file 1472-6947-14-70-S1.pdf]

Additional file 1. Assessment elements of the medical patient (n=33)

	**Assessment elements**	**Performed yes/no**
**Primary survey**	**Airway**	
	Airway assessment	
	**Breathing**	
	Assessment of spontaneous breathing	
	Assessment of breathing rate (estimated)	
	Assessment of breathing quality	
	Assessment of symmetry of breathing, and use of auxiliary breathing muscles	
	Assessment of cyanosis	
	**Circulation**	
	Assessment of external bleeding	
	Assessment of pulse rate (estimated)	
	Assessment of localization of pulse	
	Assessment of skin	
	**Disability**	
	Assessment of level of consciousness, as observed by distance	
	Assessment of pupil size and reaction to light	
	Assessment of strength of extremities	
**Anamnesis**	**Question formulation**	
	What symptoms have been observed?	
	When did symptoms start?	
	What make symptoms worse or better?	
	What is the character of the symptoms?	
	Localization or radiating?	
	Severity of symptoms?	
	Duration of symptoms?	
	Allergies?	
	Medical history?	
	Present medication?	
	Last oral intake, Elimination?	
	Events preceding the symptoms?	
**Secondary survey**		
	Breathing rate	
	Pulmonary auscultation	
	Oxygene saturation	
	Pulse rate	
	Blood pressure	
	Glasgow coma scale	
	Body temperature	
	Level of pain, Visual Analogic Scale (VAS)	
